# The role of MCP-1-CCR2 ligand-receptor axis in chondrocyte degradation and disease progress in knee osteoarthritis

**DOI:** 10.1186/s40659-015-0057-0

**Published:** 2015-11-17

**Authors:** Yuan-kun Xu, Yan Ke, Bin Wang, Jian-hao Lin

**Affiliations:** Arthritis Clinic and Research Center, Peking University People’s Hospital, No. 11, Xizhimen South Street Xicheng District, Beijing, China

**Keywords:** MCP-1, CCR2, Osteoarthritis, Chondrocyte, Rat, Mouse

## Abstract

**Background:**

Osteoarthritis (OA) is a common arthritic disease and multifactorial whole-joint disease. Interactions of chemokines and OA is inadequately documented.

**Results:**

In vivo and in vitro studies were conducted to investigate monocyte chemoattractant protein 1 (MCP-1) and receptor chemokine (C–C motif) receptor 2 (CCR2) in chondrocyte degradation and cartilage degeneration. Chondrocytes from 16 OA patients and 6 normal controls were involved in this study. After stimulation of MCP-1, the expression of MCP-1 and CCR2 increased significantly (P < 0.001) and the expression of MMP-13 also increased (P < 0.05). MCP-1 stimulation also induced (or enhanced) the apoptosis of OA chondrocytes (P < 0.05). Additionally, the degradation of cartilage matrix markers (metalloproteinase 3 and 13, MMP3 and MMP13) in the culture medium of normal chondrocytes was also assessed. Furthermore, intra-articular injection of MCP-1 in mouse knees induced cartilage degradation and the CCR2 antagonist did not impede cartilage destroy in rats knees of monosodium iodoacetate (MIA) model.

**Conclusions:**

The results of this study demonstrate that the MCP-1-CCR2 ligand-receptor axis plays a special role in the initiation and progression of OA pathology. Patients with ambiguous etiology can gain some insight from the MCP-1-CCR2 ligand-receptor axis.

## Background

OA is a common arthritic disease and multifactorial whole-joint disease. It has been recently redefined as a disease that affects the whole joint, including cellular changes, structural defects and dysfunction of all the joint compartments, e.g. cartilage, bone and synovium, the etiology of the disease is not completely defined [1–3]. Despite its widespread occurrence in the aged population, the pathogenesis of OA remains largely unknown.

The normal balance between synthesis and degradation of the cartilage matrix is biased toward degradation, and it has been shown that cytokines such as interleukin-1 (IL-1), chemokines like the C–C class of the beta chemokine family and tumor necrosis factor (TNF), as well as functional changes of the chondrocytes themselves, play major roles in the process of deterioration by inducing expression of proteinases, such as those of the MMP family. MCP-1 is a member of the C–C class of the beta chemokine family and one of the key factors involved in the initiation of inflammation. It triggers chemotaxis and transendothelial migration of monocytes to inflammatory lesions by interacting with the membrane CCR2 in monocytes [4]. MCP-1 is secreted by fibroblasts, endothelial cells, vascular smooth muscle cells, monocytes, T cells, and other cell types that mediate or are involved in the influx of cells to and at sites of inflammation [5]. MCP-1 expression has been observed in a large number of tissues during inflammation-dependent disease progression, including atherosclerosis [6], arthritis [7] and cancer [4].

As shown in early studies, OA is characterized by the degradation of cartilage, as the main (or unique) cell in cartilage; the chondrocyte plays an important role in cartilage degeneration and OA disease pathology [8–10]. We hypothesized that, despite the cytokines, the chemokines alone may also mediate certain independent interactions with chondrocytes and play a role in the pathology of OA. In this study, we investigated the interaction between MCP-1 and chondrocytes and the possible role of MCP-1-CCR2 ligand-receptor axis in cartilage degradation and disease progression in osteoarthritis.

## Results

### The expression of MCP-1-CCR2 axis genes and cartilage matrix markers after stimulation of MCP-1

**Fig. 1:**
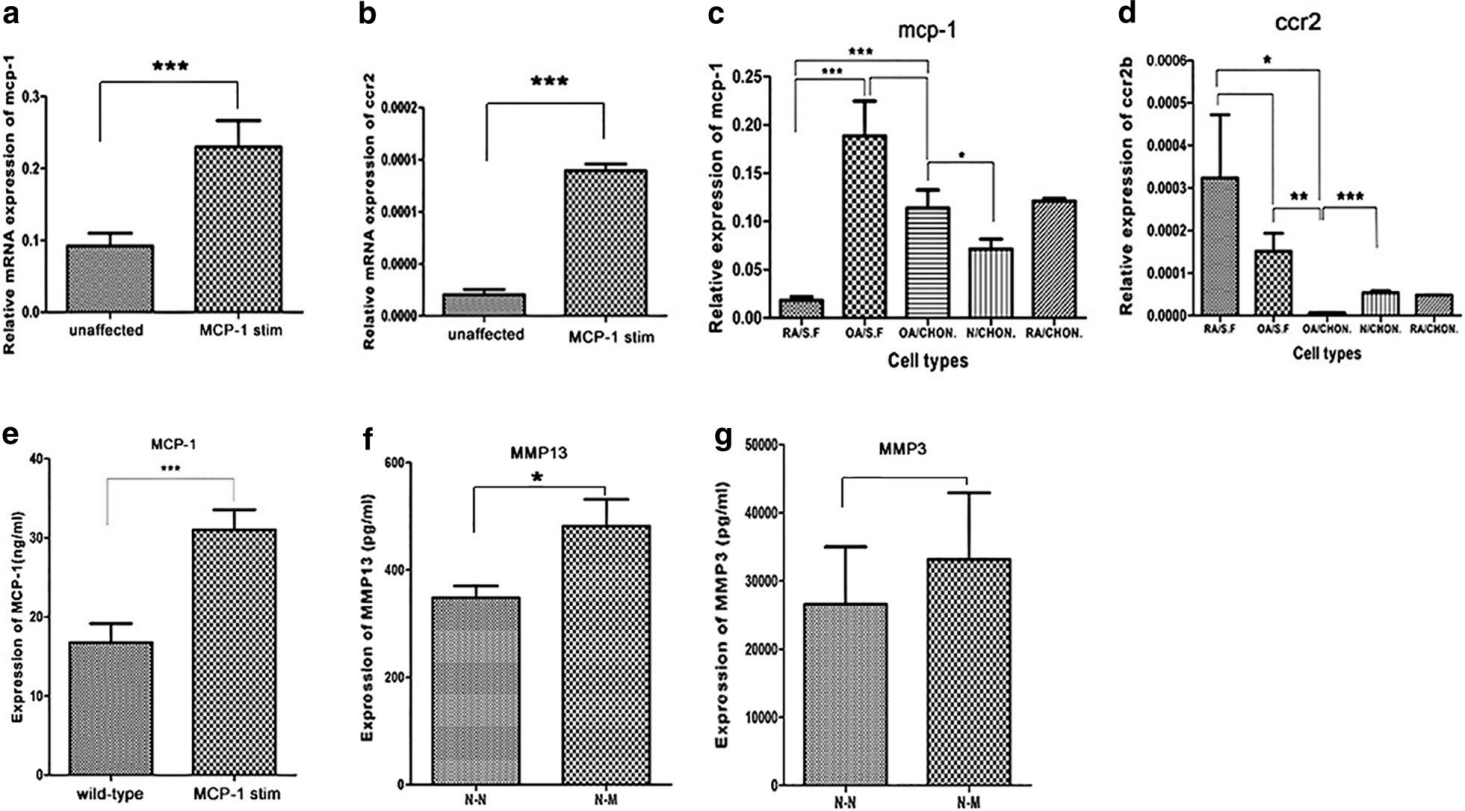
The expression of MCP-1-CCR2 axis genes and cartilage matrix markers after stimulation of MCP-1. **a**, **b** In OA chondrocytes, after stimulation of MCP-1 in culturing medium of the relative expression of MCP-1 and CCR2 mRNA increased significantly compared with unaffected OA chondrocytes; **c**, **d** the expression of Mcp-1 in OA/CHON and OA/S.F were significantly higher than RA/SF and WT/CHON. The expression of Ccr2b in OA/CHON were significantly lower than that in OA/S.F, RA/SF and WT/CHON. MCP-1 Stim, MCP-1 stimulated; RA/SF, RA synovial fibroblasts; OA/S.F, OA synovial fibroblast; OA/CHON, OA chondrocytes; WT/CHON, wild-type (normal controls) chondrocytes; RA/CHON, RA chondrocytes; and **e**, **f**, **g** after stimulation of MCP-1, the expression of MCP-1 and MMP13 protein increased significantly than unstimulated normal OA chondrocytes. MMP3 increased but there was not significantly higher than unstimulated normal chondrocytes. MCP-1 Stim, MCP-1 stimulated OA chondrocytes; N–N, unstimulated wild-type (normal controls) chondrocytes; N–M, MCP-1 stimulated wild-type (normal controls) chondrocytes. *P < 0.05, **P < 0.01, ***P < 0.001

Initially, we confirmed the presence and level of MCP-1 and CCR2 mRNAs in cultured human articular chondrocytes [11, 12]. In a total of 11 paired samples from OA patients, with or without the stimulation of MCP-1, we observed statistically significant (P < 0.001) increased expression of MCP-1 and CCR2 in stimulated chondrocytes (MCP-1 stim group) compared to the unstimulated controls (unaffected group) in each paired group (Fig. 1a, b). A comparative study of MCP-1 and CCR2 expression in a variety of chondrocytes and synovial fibroblast cells from OA, RA patients and healthy young patients was conducted. We observed that OA synovia fibroblasts had the highest expression of MCP-1; while CCR2 was mostly expressed in RA synovial fibroblasts (Fig. 1c, d).

We measured the level of chemokine protein MCP-1 secreted by chondrocytes in response to MCP-1 itself in OA and wild-type (normal controls) chondrocytes. Three days after stimulation of MCP-1 in chondrocytes from OA patients, detectable amounts of MCP-1 protein were observed and the expression of MCP-1 was higher in stimulated samples than in unstimulated controls in each pair of groups (Fig. 1e). While both MMP3 and MMP13 levels were elevated in response to MCP-1 stimulation in MCP-1 stimulated wild-type chondrocytes (N–M group) compared with unstimulated wild-type chondrocytes (N–N group), only MMP13 reached statistical significance (P < 0.05) (Fig. 1f, g).

### Chondrocyte apoptosis assessment and the expression of CCR2 in OA chondrocytes

**Fig. 2:**
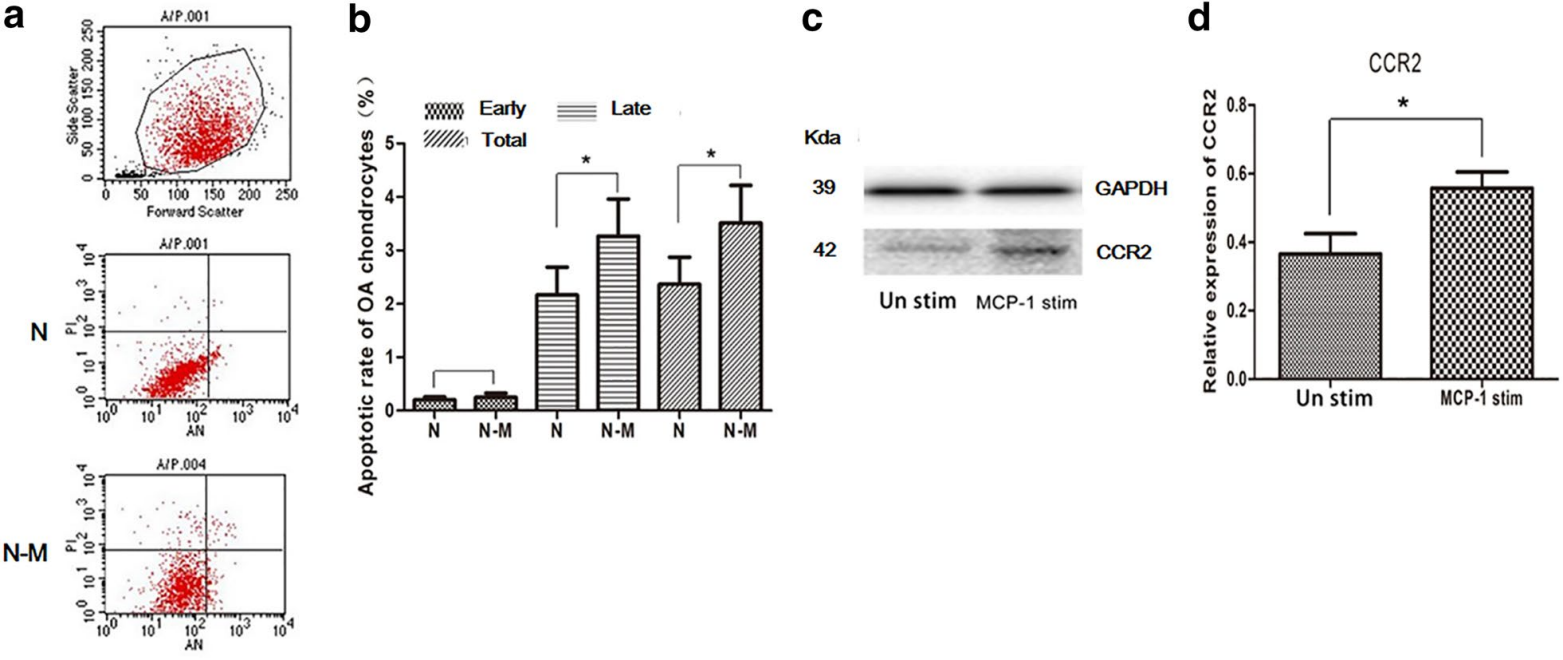
Chondrocyte apoptosis assessment and the expression of CCR2 in OA chondrocytes. **a** MCP-1 stimulation of wild-type (normal controls) chondrocytes resulted in an increased rate in secondary necrosis. *Lower left* quadrant represents viable cells, the *lower right* quadrant are chondrocytes in an early apoptotic state, the *upper right* quadrant are chondrocytes in the late apoptotic state and the *upper left* quadrant is chondrocyte necrosis. N, unstimulated wild-type (normal controls) chondrocytes; N–M, MCP-1 stimulated wild-type (normal controls) chondrocytes; **b** the variance of early apoptotic state between the two subgroups was not significant and the variance in secondary necrotic rate and both was statistically significant; and **c**, **d** MCP-1 stimulation in OA chondrocytes resulted in a significant increase in the expression of CCR2. Wild-type (normal controls), unstimulated OA chondrocytes; MCP-1, MCP-1 stimulated OA chondrocytes. *P < 0.05

To measure the rate of apoptosis by Annexin-V staining, we observed both stimulated and unstimulated OA had similar rates of apoptosis; however, the rate of secondary necrotic apoptotic chondrocytes was statistically significant higher in the stimulated compared with the unstimulated controls in each paired group (Fig. 2a, b). To quantify the expression of CCR2 protein, western blot analysis was performed. Results of western blot analysis revealed that the expression of CCR2 was lower in wild-type (normal controls) OA controls; however, stimulation of OA chondrocytes with MCP-1 enhanced the expression of CCR2 in OA chondrocytes (Fig. 2c, d).

### Micro-CT analysis of subchondral bone changes in rats

**Fig. 3:**
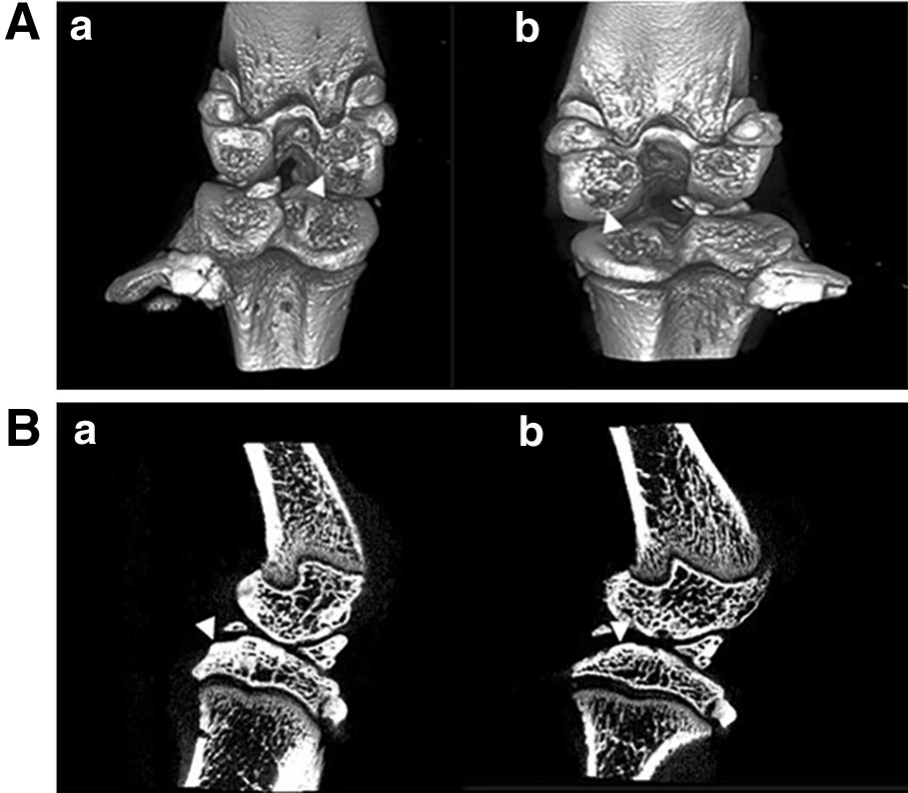
Micro-CT analysis of subchondral bone changes in rats. **A** 3D view of knee joint cartilage surface, severe cartilage damage existed in both MIA injected (*a*) and contralateral control (*b*) knees; **B** cartilage lost and subchondral bone osteosclerosis were severe in both femoral condyle and tibia in MIA injected (*a*) and contralateral (*b*) knees

According to micro CT observation, there were no significant changes in bone surface density (BV/TV), bone volume density (BS/BV), trabecular thick (TREB-THI), trabecular bone number (TREB-N), and trabecular space (TREB-S) in the whole region of the two subgroups [CCR2 antagonist injection (MIA) and contralateral physiological saline] at 2 and 6 weeks after the last CCR2 antagonist injection (Table 1). The CCR2 antagonist did not affect the parameters of the whole subchondral areas of femoral condyles and tibia, all the micro-CT analysis parameters can not reach a statistical difference between the pairs of each groups at each time point. The damage to the cartilage and subchondral bone of both experimental and contralateral knees was obvious and serious (Fig. 3A, B).

**Table 1:** The parameter of micro-CT analysis of the rats knees (each pair of subgroup n = 6)

		BV/TV (%)	BS/BV (mm^−1^)	TREB-THI (mm)	TREB-N (mm^−1^)	TREB-S (mm)
Femoral condyle						
2 week						
L		52.7 ± 6.8	17.825 ± 1.741	0.113 ± 0.011	4.651 ± 0.221	0.102 ± 0.019
R		53.2 ± 4.9	17.512 ± 1.500	0.115 ± 0.010	4.635 ± 0.242	0.101 ± 0.014
6 week						
L		71.5 ± 5.4	11.585 ± 1.220	0.174 ± 0.019	4.113 ± 0.151	0.069 ± 0.011
R		74.5 ± 4.3	11.028 ± 0.967	0.183 ± 0.017	4.089 ± 0.170	0.062 ± 0.009
Tibial						
2 week						
L		53.1 ± 5.1	18.411 ± 1.733	0.109 ± 0.011	4.855 ± 0.289	0.097 ± 0.013
R		53.6 ± 6.1	18.884 ± 2.003	0.107 ± 0.011	5.000 ± 0.112	0.093 ± 0.014
6 week						
L		75.6 ± 4.8	11.108 ± 1.016	0.181 ± 0.017	4.180 ± 0.177	0.058 ± 0.011
R		76.1 ± 6.0	11.274 ± 1.178	0.179 ± 0.018	4.262 ± 0.120	0.056 ± 0.013
Femoral condyle and tibial						
2 week						
L		52.6 ± 5.7	18.092 ± 1.568		9.506 ± 0.481	
R		53.4 ± 5.0	17.963 ± 1.365		9.634 ± 0.295	
6 week						
L		73.9 ± 5.4	11.233 ± 1.165		8.293 ± 0.322	
R		75.1 ± 4.8	11.117 ± 0.999		8.351 ± 0.272	
Patella						
2 week						
L		78.0 ± 8.4	28.159 ± 41.708	0.159 ± 0.079	4.291 ± 0.552	0.047 ± 0.024
R		80.6 ± 7.1	9.702 ± 2.143	0.214 ± 0.043	3.847 ± 0.481	0.049 ± 0.012
6 week						
L		89.4 ± 4.3	6.944 ± 1.695	0.305 ± 0.083	3.074 ± 0.614	0.033 ± 0.008
R		89.4 ± 2.2	24.565 ± 43.152	0.244 ± 0.113	10.852 ± 18.961	0.028 ± 0.013

Scan parameters of the femur condyle, tibia and patella (X ± SEM)

### Joint pathology scores in mice and rats

**Fig. 4:**
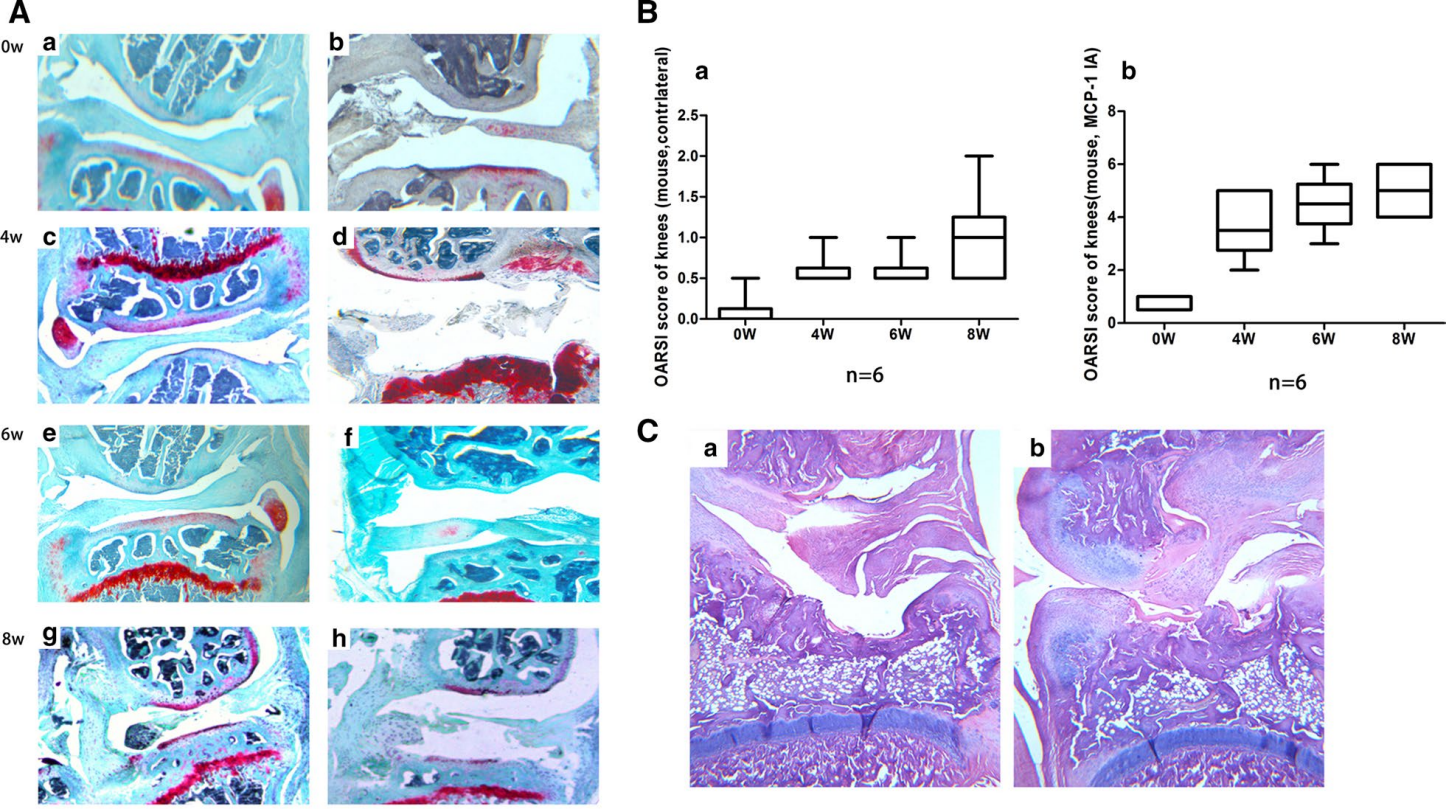
Joint pathology scores in mice and rats. **A** Safranin-O photomicrographs displaying a variety of OA severity and semi-quantitative scores. *First score* represents femoral condyle, *second score* is tibia; *a* 0, 0; *b* 0.5, 0; *c* 0.5, 2; *d* 2, 4; *e* 1, 0.5; *f* 2, 4; *g* 1, 2; *h* 4, 6. Among these, *a*, *c*, *e*, *g* shows the contralateral knees and *b*, *d*, *f*, *h* shows the MCP-1 intra-articulate injection knees. **B** The OARSI semi-quantitative scores of knees of each pairs of two subgroups at each time point; *a* scores of contralateral knees, despite some individuals with relatively high score (<2), most of the scores were low and stable; *b* scores of MCP-1 intra-articulate injection knees increased over time with some knees reaching the maximum score of 6. The rate of increase plateaued in later time-points. **C** HE slides of rats’ knee joints, articular cartilage was almost completely lost in both experiment and contralateral knee at 2 week after CCR2 antagonist or physiological saline injection. *a* The lateral tibia plateau of contralateral knee; *b* medial tibia plateau of experimental knee following CCR2 antagonist intra-articular injection. The cartilage degeneration score of Both **A** and **B** reached the highest grade of 5, at 2 weeks after CCR2 antagonist or physical saline injection

0, 4, 6, and 8 weeks following intra-articular injection of MCP-1 and 0.1 % BSA, semi-quantitative scoring of histological assessments of both lateral knee joints was made according to the OARSI initiate [13]. We can obviously see that the MCP-1 induced more serious knee joint damage than the contralateral control knee in the Safranin-O photomicrographs scoring results. 2 and 6 weeks after CCR2 antagonist injection, we examined the effect by H & E staining of rats’ knee joints (Fig. 4A, B). Cartilage degeneration score parameters described by the OARSI initiate [14] were acquired and counted. After the intra-articulate injection of MIA and CCR2 antagonist or physiological saline, the cartilage degeneration score of each knee of all slides reached the maximum grade of five (Fig. 4C). The variance between the experimental and contralateral knees were not statistically significant.

## Discussion

Despite the widespread prevalence of OA, its etiology is still unknown. Currently, known OA risk factors are not fully and clear accordant with the clinical OA etiologies. There have been many previous studies focused on the roles of cytokines and chemokines in the pathological process of OA [15].

MCP-1 is one of the chemokines which is involved in osteoarthritis, the ligand is CCR2. MCP-1 is believed to play a key role in the inflammation process [16]. Whether OA is caused by inflammatory changes or simply because the biomechanics of human joint abnormalities is contentious at present [17]. However,the classification of OA as a kind of arthritis and having some inflammation in joints is without much argument. Whether systemic or localized inflammation in focal joints is strongly associated with the origination and severity of OA, especially in those patients with normal joint mechanism, without previous trauma in/near focal joints, and with the exclusion of other risk factors, cannot be easily recognized. The role of MCP-1 in focal joints as risk factor of OA was unconfirmed. Our work focused on the role of MCP-1 as a key mediator of focal, not systemic, inflammation [18].

In our present study, we confirmed that MCP-1 operates in a positive feedback mechanism in wild-type (normal controls) and OA chondrocytes. Our experiments showed that increased MCP-1 promoted apoptosis while simultaneously inhibiting the proliferation of wild-type (normal controls) and OA cartilage cells under normal culture conditions. From our quantitative data, we determined that elevated MCP-1 levels promoted the pathogenesis of OA more so than other joint disorders, such as RA. Furthermore, we observed that an increase in MCP-1 levels in our culture system also resulted in an increase in its ligand, CCR2. Additionally, the stimulation of MCP-1 expression in wild-type (normal controls) chondrocytes resulted in the increased expression of degeneration proteins, MMP3 and MMP13, suggesting that MCP-1 attracted macrophages and other inflammatory cells to the joint and elicited an effect on cartilage cells at the same time. Through the results in our present study, we speculate that MCP-1 expedites the damage of cartilage by enhancing the apoptosis of chondrocytes while inhibiting their proliferation. Furthermore, we believe that MCP-1 induced degenerative changes in wild-type (normal controls) cartilage cells under our culture conditions.

Similar to our cell culture experiments, we observed inflammation and cartilage damage in our in vivo study after intra articular injection (IA) of MCP-1 in the knee joints of mice. The joint pathology changes induced by MCP-1 were significantly worse compared to the contralateral control injected knees. We hypothesized that treatment of an MIA rat model with a CCR2 antagonist would improve the OA observed. However, we observed no significant improvement in the pathology scores in rats and the micro CT analysis in the whole knee joint was insignificant. While we confirmed the destructive effects of MIA injection into knee joints, we encountered setbacks with intra-articular injection. Aggressive subchondral bone lesions form the basis for macroscopic evaluation [19, 20]. We would keep focus on developing structure-modifying treatments (disease-modifying OA drugs) by focusing on the CCR2 antagonist. Since there had been successful endeavors in system research of animal studies [21].

Modeling OA for research is rather complicated. While age has been confirmed to be a risk factor of OA; presently, we lack a suitable marker for diagnosis of OA disease [22]. Elevated levels of chemokines in serum in elderly individuals have been observed [23] and to quantify the degree of MCP-1 and CCR2 increase in human tissue samples, we used samples from younger, unaffected patients as a control. It is possible that we have underestimated the degree of MCP-1 increase in elderly patients with OA since our control samples were obtained from tumor patients and MCP-1 is overexpressed in many tumors [4].

## Conclusions

Taken together, the present study suggests that MCP-1 induces the degradation of wild-type (normal controls) and OA chondrocytes. These effects were embodied in the inhibition of proliferation, induction of degeneration and apoptosis of the wild-type (normal controls) and OA chondrocytes. The damaging effects of MCP-1 on cartilage cells may be due to a positive feedback mechanism. Based on the results of our present study, we infer that MCP-1 has a special role in the pathogenesis of OA and requires further investigation to delineate the MCP-1 pathway.

## Methods

### Patients

Articular cartilage and synovial membrane specimens were obtained from 18 OA (mean age 38 years, range 18–65 years), 4 RA patients (mean age 64 years, range 48–74 years) and 6 wild-type (normal controls, mean age 43 years, range 34–53 years) who were undergoing total joint replacement at Peking University, People’s hospital, Beijing. All patients met the American College of Rheumatology criteria for OA (Altman R.1986), RA (2009 ACR/EULAR) [24]. Wild-type (normal controls) (normal) human cartilage was collected from the tibial plateau of 6 patients with practically no evidence of knee joint destruction who were undergoing joint replacement because of femoral or pelvic malignant tumor. All samples were obtained with informed consent from the patients and the study protocol was approved by the ethics committee of Peking University People’s Hospital.

### Chondrocyte and synovial fibroblast isolation

Cartilage was minced and digested by rotating overnight at 37 °C in Dulbecco’s modified Eagle’s medium (DMEM; Gibco, Life Technologies, NY, USA) containing 1 mg/ml of bacterial collagenase II (Gibco). The cells released by enzymatic digestion were filtered through a sterile nylon strainer (Coring, NY, USA), washed, and centrifuged. The pellet was seeded into the six cm diameter Petri dish (Coring) at a density of 4 × 10^5^ cells and cultivated at 37 °C in a humidified atmosphere of 5 % CO_2_. The culture medium used was DMEM containing 10 % heat-inactivated fetal calf serum (Gibco), 100 units/ml penicillin (Gibco), and 100 mg/ml streptomycin (Gibco). Medium was replaced every week and cells were used between the second and third passages.

Tissues of Synovial membrane from OA and RA patients were minced and digested in DMEM containing 1 mg/ml of bacterial collagenase I (Gibco) for 3 h and then filtered and centrifuged the same as chondrocytes. The pellets was recognized as synovial fibroblasts, then seeded and cultivated at a density of 4 × 10^5^ cells, the culture medium employed the same as chondrocytes, and cells were used between the second and third passages.

### Enzyme-linked immunosorbent assay (ELISA)

After the medium was changed, cell monolayers were incubated with or without stimulation with recombinant human MCP-1 (California Bioscience, Coachella, CA) at the indicated concentrations of 20 ng/ml for 72 h. Supernatants were then collected and stored at −70 °C until used. MCP-1, collagen II and MMP3, MMP13 concentrations in the culture supernatants were evaluated with commercial ELISA kits, according to the instructions of the manufacturer. The ELISA kits for this study were as follows: MCP-1(438807, LEGEND MAX, Biolegend, San Diego, CA 92121, USA), 7.8–500 pg/ml; Collagen II (SEA572Hu, Cloud-Clone Corp. Huston, TX 77082, USA), 46.88-3000 pg/ml; MMP3 (SCA101Hu, Cloud-Clone Corp. Huston, TX 77082, USA), 41.2–60,000 pg/ml; MMP13 (SCA099Hu, Cloud-Clone Corp. Huston, TX 77082, USA). 54.9–160,000 pg/ml.

### Reverse transcriptase–polymerase chain reaction (RT-PCR)

Total RNA was extracted from the chondrocytes using the single-step method (TRIzol Reagent, Invitrogen, Carlsbad, CA, USA). First-strand complementary DNA (cDNA) was synthesized in a 10-μl reaction mixture by incubation at 37 °C for 5 min. The resulting cDNA (5 μl) was subjected to PCR using SYBRGREEN (TOYOBO, OSAKA, JAPAN) and the specific primers for the chemokines, MCP-1, CCR2, collagen II and GAPDH were showed as follow: MCP-1: Sense ATAGCAGCCACCTTCATTCC, Antisense TTTCCCCAAGTCTCTGTATCT; CCR-2b: Sense GCGGAATCTTCTTCATCATCCTC, Antisense CCTCTTCTTCTCGTTTCGACACC; GAPDH: Sense CCACCCATGGCAAATTCCATGGCA, Antisense TCTAGACGGCAGGTCAGGTCCACC.

### Flow cytometric analysis

We harvested and washed the cells, and resuspended the cells in 1 × annexin-binding buffer, added 5 μl Alexa Fluor 488 annexin V and 1 μl 100 μg/ml PI working solution (V13241, MOLECULAR PROBES, invitrogen, Eugene, OR 97402, USA) to each 100 μl of cell suspension and incubated the cells at room temperature for 15 min. After incubation, 400 μl 1 × annexin-binding buffer was added and gently mixed, while keeping the samples on ice. The stained cells were analyzed by flow cytometry, measuring the fluorescence emission at 530 and 570 nm using 488 nm excitation.

### Western blot

Protein samples were prepared by mixing sample with LDS Sample Buffer (Life Technologies, Carlsbad, CA, USA) and then boiled at 95 °C for 5 min. Proteins were separated on a NuPAGE 4–12 % Bis–Tris Gel (Life Technologies) and electro-transferred onto polyvinylidene difluoride membranes. The membranes were blocked with 5 % non-fat milk for 2 h at room temperature in Tris-buffered saline containing 0.5 % Tween-20 (TBST). The membranes were then rinsed twice with TBST and incubated with rabbit polyclonal to CCR2 (dilution of 1:2000, ab21667, Abcam, Shanghai, China) overnight at 4 °C, and then washed and incubated with corresponding goat anti-Rabbit antibody at room temperature for 1 h. Proteins were visualized via chemiluminescence with ImageQuant 350 (GE HEALTHCARE, Piscataway, NJ, USA).

### Joint pathology in mice and rats

The procedures used in this study were approved by the ethics committee of Peking University People’s Hospital. Animals were provided with living conditions, food, and housing consistent with the approved animal care operating procedures.

### Rats

Male Sprague–Dawley rats (175–200 g; Vital River Laboratory, Beijing China) were allowed to acclimate to the facility for 7 days. Rats were anesthetized with 10 % Chloral hydrate (H20064279, Peking University People’s Hospital) and given single intra-articular injection of 0.5 mg of MIA (Sigma, St. Louis, MO, USA) through the infrapatellar ligament of the knee. MIA was dissolved in physiologic saline and administered once in a volume of 25 µl using a 27-gauge, 0.5-inch needle in both knees. The right knee was the experimental, injected with 25 µl of CCR-2 antagonist (Sigma), with the concentration of 50 ng/ml [25, 26] once a week, for 4 weeks and the contralateral (left) knee was treated as a control knee which received an injection of physiological saline. The first injection of CCR-2 antagonist or physiological saline was given 3 days after the injection of MIA. The amount of MIA injected into the joint had been determined in a dose–response study [27] and we finished the intra-articulate injection of MIA in an amount of 0.25 mg/joint.

### Micro-CT analysis of subchondral bone changes in rats

Prior to histological sectioning, all rat knee joints were scanned using a micro-CT system (Inveon MM system, Siemens, Munich, Germany) with the following parameters: 360 projections over 360° increment, 600 ms exposure time averaged per projection, voltage of 80 kV, current of 500 μA, voxel size of 9.08 μm, and a scan time of approximately 20 min per knee. The reconstructed data sets were examined using three-dimensional data analysis software (Inveon Research Workplace, Siemens, Munich, Germany).

### Histology

Tissue samples were prepared for light microscopy using standard procedures. Briefly, samples were fixed in 10 % phosphate-buffered formalin and subsequently decalcified in 5 % formic acid for 72 h. Frontal section joints were routinely processed to HE slides and examined under the light microscope. The joint degeneration was assessed with the OARSI cartilage degeneration score in histological assessments of the rats [14].

### Mice

Male C57Bl/6 (Vital River Laboratory) mice aged 10 weeks were used in all experiments. The right knee joints of the mice were injected once a week, for 3 weeks, intra-articularly through the patellar ligament, with a 6-μl solution of MCP-1 (CB500038, California Bioscience, Coachella, CA, USA) at a concentration of 25 ng/ml. The dissolving agent was 0.1 % BSA, according to the manufacturer. At the same time, the left control knee joint was injected with 6 μl of 0.1 % BSA. Two mice, injected with MCP-1, were sacrificed by cervical dislocation after an inhalation of ibuprofen at two time-points 21 and 42 days, after intra-articular injection. Histological assessments of knee joints in the mouse were adopted from the OARSI histopathology initiative [13].

### Statistical analysis

Statistical analysis was performed with SPSS 21.0 (IBM SPSS, Chicago, IL, USA). Paired/unpaired *t* test was used to analyze significant difference between control and experimental groups. All data, including Micro CT, was presented as the mean ± SEM. P < 0.05 was considered statistically significant. The results of ELISA were assayed by regression analysis, Curve Expert professional 2.2 (Daniel G. Hyams) was used to draw the best standard curve. All the graphs were produced by Graphpad Prism 5 (GraphPad Software, La Jolla, CA, USA).
